# The role of a specialized urethral catheter in early detection of intra-abdominal hypertension: a case report

**DOI:** 10.1093/jscr/rjae653

**Published:** 2024-10-17

**Authors:** Dia R Halalmeh, Neha Aftab, Mohamed Hussein, Yusuf Ansari, Hutton White, Phillip Jenkins, Leo Mercer, Patrick Beer, Gul Sachwani-Daswani

**Affiliations:** Department of Trauma and Acute Care Surgery, Hurley Medical Center, 1 Hurley Plaza, Flint, MI, 48503, United States; Michigan State University College of Human Medicine, 220 Trowbridge Rd, East Lansing, MI, 48824, United States; Department of Trauma and Acute Care Surgery, Hurley Medical Center, 1 Hurley Plaza, Flint, MI, 48503, United States; Michigan State University College of Human Medicine, 220 Trowbridge Rd, East Lansing, MI, 48824, United States; Department of Emergency Medicine, Homer Stryker MD School of Medicine, Western Michigan University, 300 Portage St, Kalamazoo, MI, 49007, United States; College of Science and Technology, Temple University, 13th St, Philadelphia, PA, 19122, United States; Ascension Genesys Hospital, 1 Genesys Pkwy, Grand Blanc Twp, MI, 48439, United States; Detroit Medical Center (DMC)/Wayne State University (WSU), 4201 St Antoine, Detroit, MI, 48201, United States; Texas Tech University Health Science Center, 2500 Broadway W, Lubbock TX, 79409, United States; Department of Trauma and Acute Care Surgery, Hurley Medical Center, 1 Hurley Plaza, Flint, MI, 48503, United States; Michigan State University College of Human Medicine, 220 Trowbridge Rd, East Lansing, MI, 48824, United States; Department of Trauma and Acute Care Surgery, Hurley Medical Center, 1 Hurley Plaza, Flint, MI, 48503, United States; Michigan State University College of Human Medicine, 220 Trowbridge Rd, East Lansing, MI, 48824, United States

**Keywords:** intra-abdominal hypertension, abdominal compartment syndrome, burns, fluid resuscitation, IAP monitoring, TraumaGuard catheter

## Abstract

Intra-abdominal hypertension (IAH) and abdominal compartment syndrome (ACS) impact morbidity and mortality in burn patients, exacerbated by extensive fluid resuscitation required for more than 20% of total body surface area burns. We report a case of a 28-year-old male with severe burns and a TBSA of 49% who presented after a fire incident. The trauma team managed the patient’s fluid resuscitation, followed by early burn debridement. A TraumaGuard catheter was used for continuous intra-abdominal pressure (IAP) monitoring. On the second day of admission, a critical IAP of 20 mm Hg was detected, indicative of impending ACS. Immediate intervention with cistracurium and increased sedation reduced the IAP to 9 mm Hg, preventing the progression to ACS. This case demonstrates the importance of routine IAP monitoring in severely burned patients to prevent ACS. Early identification and management of elevated IAP can avert the progression to ACS and reduce the need for more invasive interventions.

## Introduction

Abdominal compartment syndrome (ACS) and its precursor intra-abdominal hypertension (IAH) are conditions characterized by an increase in intra-abdominal pressure (IAP) [1]. IAH leads to ACS by impeding blood flow to abdominal organs, leading to multi-organ dysfunction syndrome and, subsequently, death [1]. Both conditions are prevalent in severe burn patients, especially those with extensive total body surface area (TBSA) burns that necessitate fluid resuscitation [2].

We present a case highlighting the crucial role of continuous IAP monitoring using a TraumaGuard catheter (Sentinel Medical Technologies, Jacksonville, FL) in early recognition of IAH in a burn patient. This case prevented the progression to ACS and averted the need for more invasive interventions.

## Case presentation

Our patient was a 28-year-old male who was brought to our institution with severe burns after being found in a burning house, where he had doused himself with an accelerant. He was a nonsmoker but consumed alcohol and suffered from many mental health problems. On initial presentation, he was alert and responsive with a Glasgow coma score of 15; he later became increasingly agitated and required intubation for airway protection.

**Figure 1:**
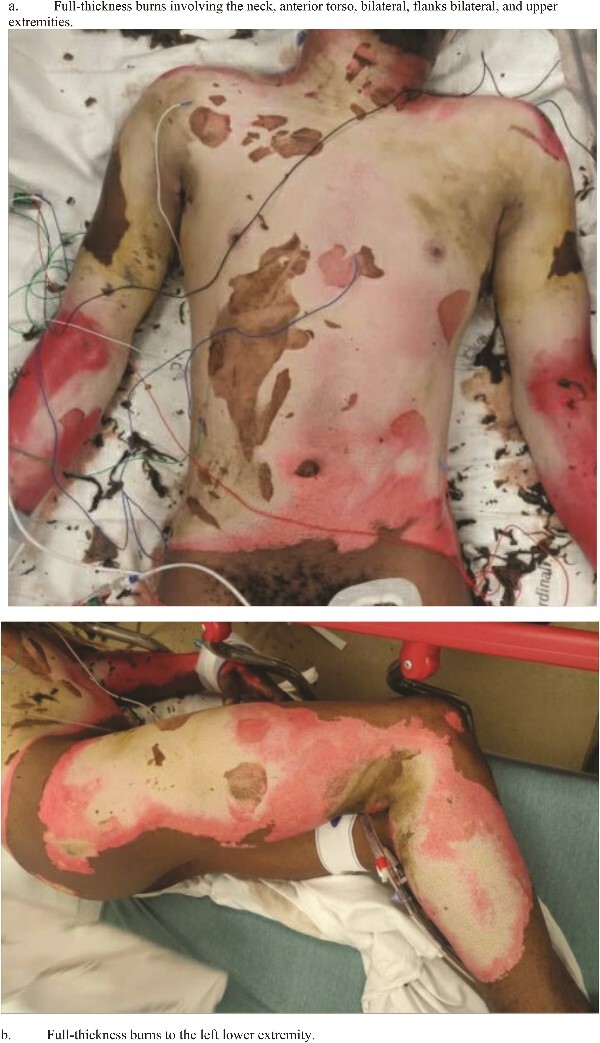
Status-post superficial debridement of burns was calculated to be 49% of TBSA. (a) Full-thickness burns involving the neck, anterior torso, bilateral, flanks bilateral, and upper extremities. (b) Full-thickness burns to the left lower extremity.

Initial assessment revealed mixed partial and full-thickness burns with sloughing noted to the entire anterior and posterior thorax, buttocks, bilateral arms circumferentially, and the right anterior thigh and the right posterior leg, totaling 38% of the TBSA. No overt signs were concerning inhalational injury (i.e. no singed nasal or facial hairs), which was excluded with bedside bronchoscopy. He was resuscitated using the Parkland formula, and the patient underwent an initial debridement in the emergency department. Upon debridement, the extension of the burn was determined to be 49% TBSA with full-thickness burns to the torso, and fluid resuscitation was recalculated (Fig. 1a and b). The patient’s total fluid requirements were 11 025 ml, of which 5512.5 ml were infused over the first hours, and the remaining 5512.5 ml were infused over the next 16 h. A TraumaGuard catheter was inserted in the trauma bay, and the patient was then admitted to the intensive care unit (ICU) for monitoring.

**Figure 2:**
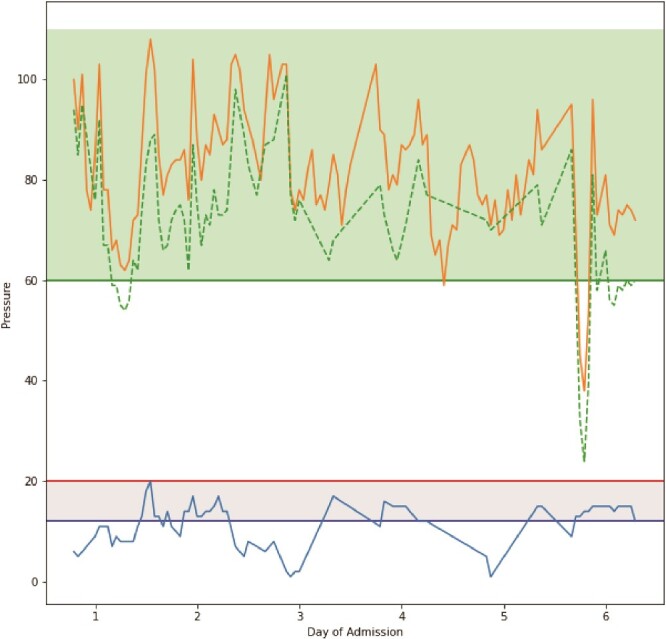
Graphical representation of pressure readings throughout the time period TraumaGuard catheter was inserted. The orange line plot represents mean arterial pressure, the dashed green line plot represents calculated abdominal perfusion pressure, and the blue line plot represents intra-abdominal pressure. Horizontal green line represents threshold for good abdominal perfusion pressure, horizontal red line represents threshold for ACS, and horizontal blue line represents threshold for IAH.

**Figure 3:**
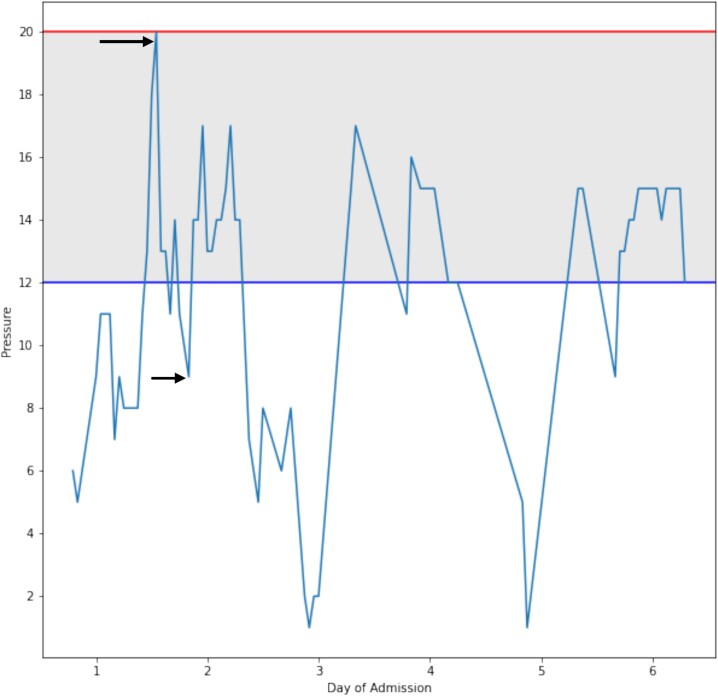
Shows a graphical representation of the change in intra-abdominal pressure that occurred during the resuscitation period. The arrows represent IAP 20 mmHg before intervention and 9 mmHg after intervention.

On Day 2, after a dressing change, readings provided by the catheter revealed an IAP of 20 mmHg, which was concerning for ACS. Concurrently, he also experienced two episodes of ventilator–dyssynchrony with elevated PCO2 (54 mmHg) and peak inspiratory pressures (PIP; 35 cm H2O) associated with concomitant rises in IAP. To control the rising IAP and rising PIP, the patient required paralysis. He received 20 mg of cisatracurium. This resulted in a normalization of the PIP. In addition, the IAP decreased to 13 mmHg and continued to decline over the day to reach 9 mmHg (Figs 2 and 3). This resulted in both the resolution of the episode and a drop in IAP. Over 4 months, the patient underwent 12 operative procedures. He required multiple excisional debridements (E&D) with cadaver skin application, followed by E&Ds that required dermal repair substitutes. He ultimately underwent split-thickness skin grafts (STSG) for all the debrided burn wounds. The development of pseudomonal infections over the debrided sites and hospital-acquired pneumonia complicated the patient’s surgical ICU course, and he required a metabolic cart to guide nutritional requirements. After adequate recovery, he was discharged to a rehabilitation facility.

## Discussion

Intra-abdominal pressure is a dynamic entity affected by changes in abdominal compliance, intra-abdominal volume, or a combination of both [1]. IAP can rise due to many causes related to the abdominal wall, abdominal cavity, or intraluminal causes [3]. Normal IAP is <5 mmHg; however, baseline IAP can be elevated in conditions like obesity and pregnancy. Abdominal perfusion pressure is calculated by subtracting IAP from MAP. Pressures above 60 mmHg are optimal for organ perfusion. In adults, an elevation of 12 mmHg is defined as IAH (10 mmHg in children), and a 20 mmHg or more increase in IAP is defined as ACS (10 mmHg in children) and requires new or worsening organ dysfunction attributable to elevated IAP [2].

Fluid resuscitation while treating severe burns can lead to edema within the abdominal cavity, putting patients at risk for developing IAP and ACS [4, 5]. Burns to the abdominal wall (or torso) can limit abdominal compliance and increase IAP. IAP can be measured via direct techniques that include an intra-peritoneal pressure transducer and indirect techniques that include intra-vesical, central-venous, rectal, and intrauterine pressures [1].

We used a novel urethral catheter, which, in addition to routine urine output measurements, continuously measured IAP via the abdominal pressure changes noted via the urinary bladder (intra-vesicle). The catheter is connected to the monitor via a transducer, allowing continuous monitoring. Continuous intra-vesical pressure monitoring, such as the TraumaGuard catheter, has some limitations in detecting secondary ACS (sACS) in burn patients [6]. One of the primary challenges is the reduced reliability of urine output as an indicator of perfusion and resuscitation status. sACS involve a rapid increase in IAP, which can compromise renal perfusion and decrease urine output [7]. This can mislead clinicians into administering additional fluid, further exacerbating the situation.

A definite advantage of the TraumaGuard catheter is the ability to immediately obtain IAP readings once the catheter is placed within the urinary bladder and its patented balloon is inflated. Ball and Kirkpatrick [8] showed that larger priming volumes can artificially increase the measured IAP. Therefore, it is recommended to use a priming volume of <75 cm^3^ to minimize these inaccuracies. Despite this adjustment, the technique requires careful calibration and interpretation by clinicians to ensure accurate readings [6]. While continuous intra-vesical pressure monitoring can provide valuable real-time data, it should be complemented by clinical oversight and other diagnostic techniques.

While surgical decompression represents a definitive treatment strategy for ACS [9], many conservative approaches like intraluminal decompression, drainage of abdominal fluid, balancing fluid input and output, or sedation and paralysis can be used [1]. This requires continuous IAP monitoring in high-risk patients, early recognition, and early intervention to prevent progression to ACS. Using a urethral catheter that is equipped with an electronic transducer in our patient allowed us to continuously monitor IAP, recognize the development of IAH, and utilize early interventions to prevent the progression to ACS. Integrating this technology with clinical assessments and adhering to established protocols can enhance patient outcomes.
